# Identification of antibiotic induced persister cells in *Streptococcus agalactiae*

**DOI:** 10.1371/journal.pone.0303271

**Published:** 2024-06-26

**Authors:** Nanna Boll Greve, Hans-Christian Slotved, John Elmerdahl Olsen, Line Elnif Thomsen

**Affiliations:** 1 Faculty of Health and Medical Sciences, Department of Veterinary and Animal Sciences, University of Copenhagen, Frederiksberg C, Denmark; 2 Department of Bacteria, Division of Infectious Disease Preparedness, Parasites and Fungi, Statens Serum Institut, Copenhagen S, Denmark; Hawassa University College of Medicine and Health Sciences, ETHIOPIA

## Abstract

Antibiotic persistence is a phenomenon, where a small fraction of a bacterial population expresses a phenotypic variation that allows them to survive antibiotic treatment, which is lethal to the rest of the population. These cells are called persisters cells, and their occurrence has been associated with recurrent disease. *Streptococcus agalactiae* is a human pathobiont, able to cause invasive infections, and recurrent infections have been reported to occur in both newborns and adults. In this study, we demonstrated that *S*. *agalactiae* NEM316 can form persister cells when exposed to antibiotics from different classes. The frequency of persister cell formation was dependent on bacterial growth phase and the class of antibiotics. The ability to form persister cells in response to penicillin was shown to be a general trait among different clinical *S*. *agalactiae* isolates, independent of sero- and sequence-type. Taken together, this study shows the existence of antibiotic tolerant *S*. *agalactiae* persister cells, which may explain why this bacterial species frequently persists after treatment of invasive infection and can be associated with recurrent disease.

## Introduction

Phenotypic diversity within a genetically identical bacterial population allows a small fraction of cells to survive lethal treatment with antibiotic [1–4]. The surviving fraction of cells are known as persister cells or persisters. They are genetically identical to the sensitive part of the population and rely on a phenotypically reversible switch between being growing cells and entering a physiologically dormant state for the survival [4]. Persister cell formation results in biphasic killing kinetics during antibiotic treatment. An initial steep slope reflects the death of the sensitive part of the population, followed by a slower rate of killing, until it reaches a plateau, revealing the surviving persister fraction. The persister phenomenon has been described in several species, including *Escherichia coli*, *Salmonella* Typhimurium, and *Pseudomonas aeruginosa* [5–7].

Antibiotic persistence has been observed after treatments with antibiotics of various classes [1]. It is an example of triggered persistence, which is an active response, induced not only by antibiotic treatment, but by other stress factors as well, including starvation, extreme pH and temperatures, and DNA damage [8,9]. However, spontaneous persistence can also occur due to individual variations in the cellular molecular and biochemical processes influencing gene expression, leading to the persister state in a more stochastic manner [1,4,8]. Regardless of whether the persister state is stress-triggered or occurs spontaneously, the persister phenotype is transient, and the persisters can reinitiate growth, and give rise to a new population, which will be as sensitive to the antibiotic as the original population [1–4]. Hence, persister cells can complicate the treatment of bacterial infections, and have been associated with recalcitrance and recurrence of infections [10–13].

*Streptococcus agalactiae* (Group B Streptococci (GBS)) is the leading cause of neonatal pneumonia, sepsis, and meningitis worldwide [14]. It is a pathobiont and a commensal colonizer of the respiratory, gastrointestinal, and female genital tracts. Vertical transmission of GBS from mother to baby during labor is considered the source of early-onset neonatal disease (EOD), occurring within the first 0–6 days of life [15,16]. Efforts to prevent EOD in newborns have been put into action, with screening of pregnant women for GBS colonization and offering intrapartum antibiotic prophylaxis (IAP). But, despite the implementation of these preventive strategies, it has not been successful in eliminating EOD [17]. Furthermore, these prevention strategies have not had any effect on late-onset disease (LOD) in infants aged 7–89 days [15,18,19]. In recent years, GBS has gained recognition as a significant global health concern in not only newborns but also non-pregnant adults, particularly in the elderly population [20–22]. Recurrent GBS infections have been observed in both infants and adults, where relapse, rather than a new acquisition, have been found to be the most common reason [20,23–25].

In the current study, we hypothesize that antibiotic induced persister cells are formed when GBS is subjected to treatment with different classes of antimicrobials and that this may play a role in recurrent GBS infections. The aim of this study was to examine the ability of GBS to form a sub-population of antibiotic tolerant persister cells. We analyzed the persister cell formation during different growth phases with exposure to different classes of antibiotics. As proof of concept, we investigated whether clinical GBS isolates were able to form persister cells when exposed to penicillin, which is the most commonly used antibiotic against GBS. We show that GBS has the ability to form a sub-population of persister cells in response to treatment with different classes of antimicrobials, and that this is a common trait for all strains investigated.

## Materials and methods

### Bacterial strains and culturing

The bacterial strains used in this study consist of *S*. *agalactiae* NEM316 (ATCC12403)(European Nucleotide Archive (ENA) under accession ID AL732656), nineteen invasive strains of GBS, and three GBS carrier isolates (listed in Table 1). Clinical isolates from patients with invasive GBS were collected routinely for national surveillance purposes by Statens Serum Institut (SSI). The carriage isolates were collected as a part of a study on quality assessment of PCR tests [26]. Bacteria were grown in Tryptone Soya Broth medium (TSB) (Oxoid CM0129) or on TSB agar plates (TSA) (Oxoid CM0131) at 37 °C with shaking (180 RPM). Identification and serotyping of the GBS isolates was performed as previously described [27]. The genomic sequence data for the 22 clinical isolates have been deposited at the European Nucleotide Archive (ENA) under Bioproject no. PRJEB72270.

**Table 1:** *Streptococcus agalactiae* strains used in this study.

Strain	Serotype and Sequence Type (ST)	origin	Age	No. isolate from recurrent disease	Reference
NEM316	Serotype III, ST23	-	-	-	ATCC-12403
NB401	Serotype Ia, ST23	Invasive	0–6 days (EOD)	-	SSI
NB402	Serotype Ia, ST23	Invasive	0–6 days (EOD)	-	SSI
NB403	Serotype III, ST17	Invasive	0–6 days (EOD)	-	SSI
NB404	Serotype III, ST17	Invasive	0–6 days (EOD)	-	SSI
NB405	Serotype Ia, ST23	Invasive	0–6 days (EOD)	-	SSI
NB406	Serotype III, ST17	Invasive	0–6 days (EOD)	-	SSI
NB407	Serotype III, ST17	Invasive	0–6 days (EOD)	-	SSI
NB408	Serotype III, ST17	Invasive	0–6 days (EOD)	-	SSI
NB409	Serotype III, ST17	Invasive	0–6 days (EOD)	-	SSI
NB410	Serotype III, ST17	Invasive	20–40 years	-	SSI
NB411	Serotype III, ST17	Invasive	20–40 years	-	SSI
NB412	Serotype III, ST529	Invasive	20–40 years	-	SSI
NB413	Serotype III, ST17	Invasive	20–40 years	-	SSI
NB414	Serotype III, ST17	Invasive	20–40 years	-	SSI
NB423	Serotype IX, ST130	Carrier	30+ years	-	SSI
NB428	Serotype IX, ST19	Carrier	30+ years	-	SSI
NB429	Serotype IX, ST4	Carrier	30+ years	-	SSI
NB430	Serotype V, ST1	Invasive	60+ years	1. isolate	SSI
NB431	Serotype V, ST1	Invasive	60+ years	2. isolate	SSI
NB432	Serotype VIII, ST1	Invasive	60+ years	1. isolate	SSI
NB433	Serotype VIII, ST1	Invasive	60+ years	2. isolate	SSI
NB436	Serotype V, ST498	Invasive	60+ years	-	SSI

#### Ethical statements

All isolates were collected in accordance with relevant guidelines and regulations.

The carriage isolates were collected as a part of a study on quality assessment of PCR tests [26] which was approved by the Danish Data Protection Agency (j.nr. 2012-58-0018), and do not require approval from an ethics committee. Regarding the isolates from patients with invasive GBS: the data and samples from patients are collected routinely for national surveillance purposes, no ethical approval or informed consent from patients or guardians are required. Statens Serum Institut (SSI) which is under the auspices of the Danish Ministry of Health, have a general approval by the Danish Data Protection Agency (record number 2007-41-0229) (https://en.ssi.dk/about-us/information-about-processing-of-personal-data, accessed 22-11-2023) to publish the data.

All data on the isolates were fully anonymized before being accessed.

### Determination of minimal inhibitory concentration (MIC)

The MIC of penicillin, ampicillin, ciprofloxacin, vancomycin, and gentamicin (Sigma, Copenhagen, Denmark) was determined by micro broth dilution method. An overnight (ON) culture in TSB medium was diluted to an optical density of 0.2 at 600 nm (OD_600_) in fresh TSB medium. A 2-fold increment of each antibiotic was added to wells containing 0.2 ml of the broth, resulting in testing of susceptibility in the range from 0 to 512 μg/ml of each antimicrobial. The culture was incubated for 18 hours at 37 °C and MIC was determined as the first concentration which inhibited growth as judged by lack of turbidity.

### Test for persister cell formation by antibiotic killing assay

To obtain late-exponential cells, an ON culture was diluted to OD_600_ = 0.03 and grown to late-exponential phase (OD_600_ = 0.5). To obtain mid-exponential (OD_600_ = 0.2) cells, ON cultures were diluted to OD_600_ = 0.03 and grown to mid-exponential phase before re-diluting the culture to OD_600_ = 0.03 and grown to mid-exponential growth phase a second time. At the desired OD_600_ from each growth phase, a sample was withdrawn (T_0_) and 100-fold MIC of either penicillin (6.25 μg/ml), ampicillin (24.98 μg/ml), ciprofloxacin (100 μg/ml), vancomycin (100 μg/ml), or gentamicin (3200 μg/ml) was added to the cultures. The killing kinetics were determined by assessing colony forming units (CFU) after the antibiotic treatments at different timepoints. This was obtained by serially diluting the cultures in a 0.9% NaCl solution and plating 10 μl drops of each dilution on TSB agar plates in technical replicates. After 24 hours incubation the CFU was counted. The persister fractions were calculated as the survival percentage of CFU from the last time-point relative to CFU at T_0_. The limit of detection was defined as 10^2^ CFU/ml and bacterial count below this level were considered not detectable.

### Heritability-assay

Heritability of the persister state was tested for the penicillin- and ciprofloxacin-induced persister fractions. Surviving colonies at the last time point of the killing assays from mid-exponential phase were pooled and grow ON, diluted and grown to mid-exponential phase. A sample (T_0_) was taken, and 100-fold MIC of penicillin (6.25 μg/ml) and ciprofloxacin (100 μg/ml), respectively, was added to the cultures. The killing kinetics were determined by assessing colony forming units (CFU) after 25 hours of antibiotic treatments. The experiment was performed in biological triplicate.

## Results and discussion

### Persister cells are formed as a dynamic measure in late-exponential phase cultures of *Streptococcus agalactiae*

The main characteristic for persister cells is their ability to tolerate concentration of antibiotics above the minimal inhibition concentration (MIC). To investigate whether *Streptococcus agalactiae* (GBS) is able to form persister cells, we determined the MIC of four different classes of bactericidal antibiotics on the GBS strain NEM316 (serotype III). The MIC was determined for penicillin G (0.0625 μg/ml), ampicillin (0.25 μg/ml), ciprofloxacin (1 μg/ml), vancomycin (1 μg/ml), and gentamicin (32 μg/ml). We performed an antibiotic killing assay on GBS grown to late-exponential phase with 100-fold MIC of each antibiotic, to ensure sufficient killing of the antibiotic-sensitive part of the population, as well as any bacteria that would express a lower susceptibility than rest of the population.

**Fig 1:**
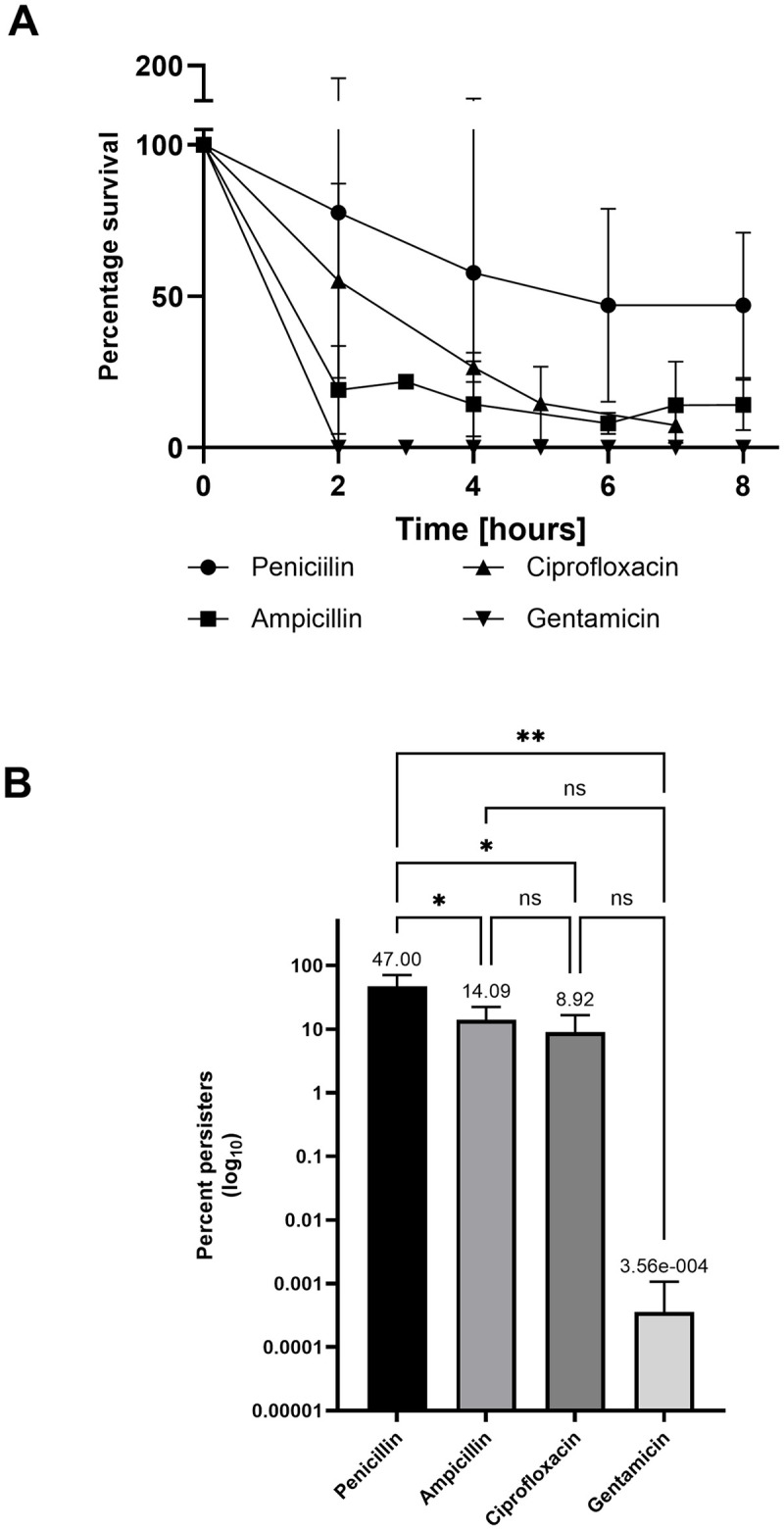
Killing kinetics and persister fraction of GBS exposed to different antibiotics in late-exponential phase. Late-exponential grown GBS strain NEM316 was challenged with 100-fold MIC of indicated antibiotics over time. (A) The percentage survival is shown for each time point, and for each antibiotic used; penicillin ●, ciprofloxacin ▲, ampicillin ■, and gentamicin▼. (B) The persister cell fraction in percentage for each antibiotic after 8 hours. The values are means of three biological replicates and error bars indicate the standard deviation. Statistical comparison of mean fraction of persister cell formation between different antibiotic treatments was done by One way Anova with Turkey correction for multiple comparisons using GraphPad Prism version 10.2.1.

The antibiotic challenge of the late-exponential phase culture resulted in a substantial β-lactam and ciprofloxacin tolerance. For these cultures, a biphasic killing pattern was obtained, which revealed a sub-population of persister cells able to survive the antibiotic treatment (Figs 1A and S1). A drastic drop in the CFU count was observed within the first 2–3 hours of the killing assay. For ampicillin the CFU count was stabilizes already after 2 hours, for ciprofloxacin after 3 hours and for penicillin after 6 hours. After 8 hours of treatment the final survival rate was 47% for penicillin (+/- 24.04%), 14.09% for ampicillin (+/-8.35%), 8.92% for ciprofloxacin (+/- 7.68%), and 0.000356% for gentamicin (+/- 0.0007%) (Fig 1B). A culture without antibiotic exposure served as growth control (S1 Fig).

The killing curve showing CFU/ml (log10) is available in S1 Fig.

Overall, we observed high persister cell frequencies and large fluctuations between the biological repetitions for the different antibiotic classes. Specifically, in the case of gentamicin, no surviving cells could be detected within the first two hours for the gentamicin treated culture in two biological replicates, whereas 0.0014% was observed in the third biological replicate after 8 hours (Figs 1A, 1B and S1). The high persister cell frequencies and large fluctuations may be because the cultures were not completely homogenous, and some of the cells had already entered stationary phase. For several other bacterial species, the highest frequency of persister cells has been reported to be formed in stationary growth phase, most likely induced by the lack of nutrients and high cell density [9,28–31]. When entering of cells into stationary phase occurs in a non-uniform manner, it will make the persister count a dynamic measure, and the antibiotic triggered persistence will be reinforced by other stress factors that also induce the persister state [9,28–30]. This suggest that our observed persister frequency may be a result of a non-uniform entering into stationary phase, and the induction to the persister state did not completely reflect antibiotic persistence, but also a response to starvation and increasing cell density. This could explain both the high fluctuation of the persister frequency between the experiments and the overall high persister fraction for all the different antibiotics tested.

### Mid-exponential phase *Streptococcus agalactiae* cultures form antibiotic induced persisters cells in response to different classes of antibiotics

To ensure that our analysis focus on only antibiotic induced persister cell formation, we chose to analyze persister cell frequencies in mid-exponential cultures. Previous studies have shown that the stationary phase triggered persister state can remain even when the cells are given the opportunity to grow, such as by diluting an stationary phase culture in fresh medium [1]. Hence, pre-existing persisters can influence the results even after several hours of growth [1,32]. Therefore, to ensure a homogenous culture and to eliminate the influence of stationary-phase carry-over persister cells that could affect the persister frequency, we grew the cultures to mid-exponential phase, re-diluted and grew again to mid-exponential phase before analyzing the persister cell levels. A culture without antibiotic exposure served as growth control (S2 Fig).

**Fig 2:**
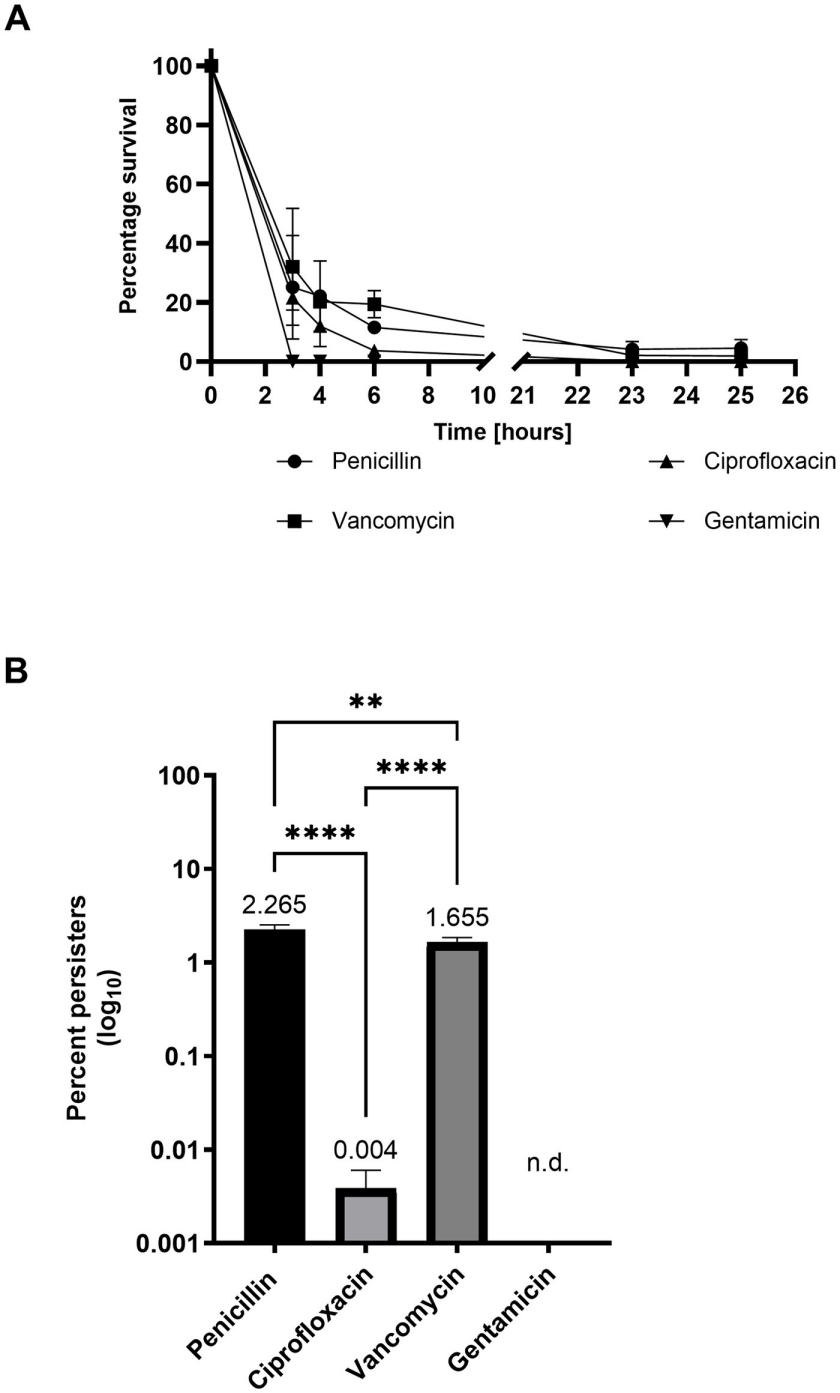
Killing kinetics and persister fraction of GBS exposed to different antibiotics in mid-exponential phase. GBS strain NEM316 was grown to mid-exponential phase twice before challenged with 100-fold MIC of indicated antibiotics over time. (A) The percentage survival is shown for each time point, and for each antibiotic used; penicillin ●, ciprofloxacin ▲, vancomycin ■, and gentamicin▼. (B) The persister cell fraction in percentage for each antibiotic after 25 hours or noted with n.d. for not detected. The values are means of three biological replicates and error bars indicate the standard deviation. Statistical comparison of mean fraction of persister cell formation between different antibiotic treatments was done by One way Anova with Turkey correction for multiple comparisons using GraphPad Prism version 10.2.1. The killing curve showing CFU/ml (log10) is available in S2 Fig.

For the penicillin, ciprofloxacin, and vancomycin treated cultures, biphasic killing kinetics could be observed. Similar to the late-exponential cultures, the majority of the cultures were killed within the first 3 hours of the assay followed by the formation of a plateau, revealing the presence of a persister fraction in the three cultures (Fig 2A). Only a small fluctuation in the persister frequency between the assays was observed this time. Again, we observed that the persister fraction varied dependent on the antibiotic used. The treatment with penicillin resulted in the highest persister subpopulation, with a persister fraction at 2.3% (+/- 0.3%), followed by vancomycin treatment resulting in a persister fraction at 1.7% (+/- 0.2%), and when treated with ciprofloxacin it resulted in a persister fraction at 0.004% (+/- 0.002%) (Fig 2B). However, this time showing a clear stabilization of the CFU counts between 23 and 25 hours (Figs 2A and S2). It was not possible to detect any surviving cells in the gentamicin treated culture (Fig 2A and 2B). But with a detection limit of 100 CFU/ml, we cannot exclude the presence of low frequency persister cells. This is supported by the results using late-exponential phase bacteria which revealed very low to not detectable levels of gentamicin induced persisters cells. This suggests that GBS can form gentamicin induced persister cells, but at low rates.

To confirm that the observed surviving fraction of cells indeed was the persister fraction, and not caused by a mutation giving a population-wide tolerance, we performed a heritability assay. The surviving colonies from either the penicillin or ciprofloxacin killing assays were exposed to a second round of either penicillin or ciprofloxacin treatment. We observed similar levels of persister cells, confirming that the persister cell phenotype was transient and not inheritable (S3 Fig).

In both mid-exponential and late-exponential phase, we could observe a variation in the persister frequency depending on the antibiotic used. If one physiological change was responsible for the persister phenomenon, it would have been expected that similar levels of persister cells would be formed, independent of the used antibiotic. Since ciprofloxacin treated GBS had a much lower persister fraction in both growth phases compared to the β-lactams and vancomycin treated, we suggest that different antibiotics might activate distinctive intracellular pathways, which promote the formation of the persister phenotype by different mechanisms. Previously published data on *E*. *coli* and *Staphylococcus saprophyticus* revealed different levels of persister cell formation dependent on the mode of action of the antibiotics used [33,34]. Interestingly, there was no apparent association between persister fractions, even when they compared antibiotics that had closely related modes of action. Furthermore, analysis of *Salmonella enterica* species suggested that different antibiotics induced persister cells at varying levels, due to differences in the bacterial response [35]. In addition, as it was not possible to detect any surviving persister cells from the gentamicin treated culture in the mid-exponential growth phase (Fig 2B), it supports our hypothesis that there is not one distinct molecular mechanism that leads to persistence. Willenborg and colleagues have previously investigated gentamicin tolerance in several streptococcus species, but only *Streptococcus suis* was found to form a persister fraction [36]. Hence, they suggested that a specific mechanism for gentamicin tolerance has evolved separately in *S*. *suis* and is not a shared genetic trait within the genus *Streptococcus*. However, our data reveal that other streptococcus species may also be able to form gentamicin induced persister cells, but the number may fall below detection limits. This reinforces the idea that multiple molecular mechanisms can lead to persister cell formation in GBS and that persisters to one drug may be different from persisters to another drug. However, further analysis is required to understand the differences between the bacterial response to antibiotics, and the specific part of that response, that leads to persister cell formation.

### Penicillin induced persister cell formation differs between various clinical *S*. *agalactiae* isolates

Next, we wanted to verify that the ability to survive antibiotic treatment by forming a persister fraction is a general phenomenon among GBS strains. We investigated 22 different clinical GBS isolates with different serotypes and sequence types (ST), isolated from neonates with early-onset disease (EOD), asymptomatic carrier, invasive disease in non-pregnant adult, or recurrent infections. All strains were grown to mid-exponential phase and sub-cultured before we performed the antibiotic killing assay with 100-fold MIC (6.25 μg/ml) of penicillin for a period of 25 hours. This concentration is above the EUCAST clinical breakpoint of 0.125 μg/ml [37].

**Fig 3:**
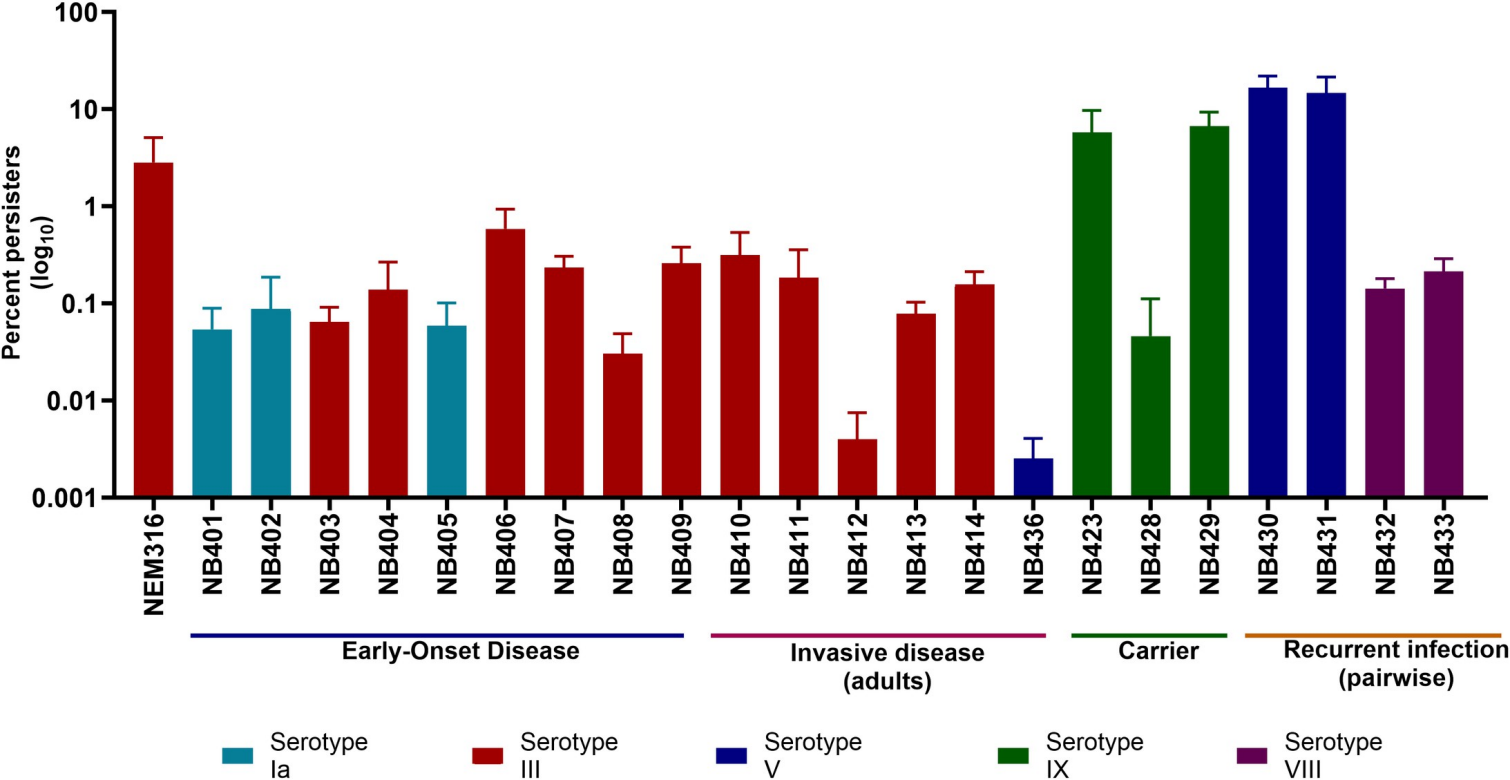
Persister cell levels of GBS clinical isolates. Clinical isolates of different GBS strains were grown to mid-exponential phase twice before challenged with 100-fold MIC of penicillin. The clinical isolates names are color-coded according to their serotype: Light blue for serotype Ia, red for serotype III, green for serotype IX, dark blue for serotype V, and purple for serotype VIII, and arranged by origin. The persister cell fraction for each strain is presented. The values are means of three biological replicates and error bars indicate standard deviation. The killing curve showing CFU/ml (log10) is available in S4 Fig.

For all the strains, this resulted in a steep killing slope followed by a plateau at the last two timepoints, revealing that all the clinical strains could form a subpopulation of persister cells surviving the penicillin treatment (Figs 3 and S4). For the two individual pairs of recurrent isolates, we observed a similar frequency of persister cells originating from the first and second isolate (Fig 3). One isolate in each pair was obtained before treatment and the other from the re-current infection, i.e. after antibiotic treatment. This result supports that persister cells, after antibiotic exposure is ceased, revert to a growing population equally sensitive to antibiotic exposure as the original population. However, we observed a substantial variation in the persister frequency between all the clinical strains, ranging from 0.004% to 16.67%.; but no pattern in relation to either serotype, ST, or origin could be observed (Fig 3).

Similar variations in persister cell frequency among various isolates have been seen in other studies. In collections of environmental and clinical *E*. *coli* isolates they observed, despite having similar MIC values, variations of four orders of magnitude in the persister cell frequency, when exposed to the same antibiotic [34,38]. Comparable large variations have also been observed when investigating persister cell formation in different clinical isolates of *Streptococcus pneumoniae* [39].

Hence, variations in persister frequency among different isolates have been seen, however, we speculated if this variation was caused by genetic differences and performed a comparative genomic analysis of the 22 GBS clinical isolates. The whole nucleotide content of the clinical strains was aligned and compared to NEM316. We made the graphical alignment of the genomes to reflect the persister frequency, arranged from highest level in the central and progressively lower values towards the outer circle (S5 Fig). This comparison revealed variability among the genomes, identifying 13 regions with notable differences. But no pattern in relation to the persister level could be observed, as some regions would be present in both high scoring persister strains as well as low scoring persister strains. Equally, missing regions could also be found in both high and low scoring persister strains (S5 Fig).

**Fig 4:**
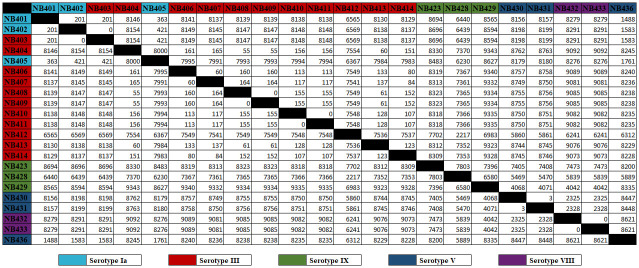
Pairwise whole-genome SNP comparison of GBS clinical isolates. Single nucleotide polymorphisms (SNPs) between the clinical isolates were detected by pairwise comparison of all isolates. These SNPs cover insertions, deletions, and substitutions of nucleotides in the genome sequence, and are quantified as the number of pairwise SNP differences. The names of the clinical isolates are color-coded according to their serotype: Light blue for serotype Ia, red for serotype III, green for serotype IX, dark blue for serotype V, and purple for serotype VIII.

However, the alignment revealed multiple smaller variations between the genomes, so we made pairwise whole-genome single nucleotide polymorphism (SNP) comparison of the strains (Fig 4). This analysis showed that despite having the same serotype, ST, and similar levels of persistence, substantial variations could be observed between the strains. For instance, there were more than 8000 SNPs identified between strain NB403 and NB413 (Fig 4). Whereas strains NB408 and NB409 had no SNPs, despite different persister cell frequencies. Hence, no clear pattern could be observed between high and low level persister frequencies and the number of SNPs.

Other studies have previously shown that antibiotic therapy and antibiotic prophylaxis can enrich the persister subpopulation over time [11,33]. Hence, the observed variations between the different clinical isolates may be explained by the history of antibiotic treatment of the individual patients. However, large variations in persister cell frequencies within *E*. *coli* isolates without any known history of drug exposure have also been observed, which indicates that variations may also arise due to other environmental stresses [38]. This highlight the complexity of bacteria’s ability to form persisters and cannot solely be explained by genomic factors. Hence, the variations in persister frequencies between the different clinical GBS isolates we have observed in this study may be explained by the history of antibiotic treatment and the host environment of individual patients.

However, our results confirm that the ability to form persister cells is a common phenomenon, but with high variety between different GBS strains, and suggests a clinical relevance of GBS persister cells.

## Conclusion

The results of the current study show that the pathobiont GBS is able to form persister cells in response to different classes of antibiotics. Our findings reveal that the persister frequency of GBS is growth-phase and antimicrobial compound dependent, which suggests the existence of different mechanisms behind antibiotic persistence. Further, all clinical isolates of GBS tested in this study were capable of forming persisters in response to penicillin treatment, however, with large variation between strains. These findings imply that the formation of persister cells is a common trait shared by various GBS strains. It is therefore strongly recommended to explore the potential association between recurrent GBS infections and antibiotic persistence.

## Supporting information

S1 Fig CFU killing curve, late-exponential. Late-exponential grown *S*. *agalactiae* strain NEM316 was challenged with 100-fold MIC of indicated antibiotics over time. The CFU/ml was calculated for each time-point, with a detection limit of 100 CFU/ml. The values are means of three biological replicates and error bars indicate the standard deviation.
(TIF)

S2 Fig CFU killing curve, mid-exponential. *S*. *agalactiae* strain NEM316 was grown to mid-exponential phase twice before challenged with 100-fold MIC of indicated antibiotics over time. The CFU/ml was calculated for each time-point, with a detection limit of 100 CFU/ml. The values are means of three biological replicates and error bars indicate the standard deviation.
(TIF)

S3 Fig Heritability assay. Surviving colonies from the first round of killing assay with (A) penicillin or (B) ciprofloxacin, has been exposed to a second round of killing using the same antibiotic. The persister cell fraction in percentage for each antibiotic after 25 hours are noted, and unpaired t-test was performed (*P* = <0.05), n.s. stands for not significant. The assay was performed in biological triplicates.
(TIF)

S4 Fig CFU killing curve, clinical isolates. Clinical isolates of different *S*. *agalactiae* strains were grown to mid-exponential phase twice before challenged with 100-fold MIC of penicillin, and the CFU/ml was calculated for each time-point. The values are means of three biological replicates and error bars indicate the standard deviation.
(TIF)

S5 Fig Genomic comparison of GBS clinical isolates in relation to persister levels. A comparative genomic analysis of all clinical isolates, organized according to persister levels. The central circle indicates the highest value, and the outer circles represent progressively lower values of persister frequency. Each circle is color-coded to reflect the persister level: Dark green for 10–19%, light green for 1–9%, yellow for 0.1–0.9%, light red for 0.01–0.09%, and dark red for 0.001–0.009%. The regions 1 to 13 highlight the areas with highest variations compared to NEM316. The figure is drawn by the Basic Local Alignment Search Tool (BLAST) Ring Image Generator (BRIG) using the genome of NEM316 as reference.
(TIF)

## References

[1] Balaban NQ, Helaine S, Lewis K, Ackermann M, Aldridge B, Andersson DI, et al. Definitions and guidelines for research on antibiotic persistence. Nature Reviews Microbiology. 2019;17(7):441–448. doi: 10.1038/s41579-019-0196-3 30980069 PMC7136161

[2] Jung S-H, Ryu C-M, Kim J-S. Bacterial persistence: Fundamentals and clinical importance. Journal of Microbiology. 2019;57(10):829–835. doi: 10.1007/s12275-019-9218-0 31463787

[3] Van den Bergh B, Fauvart M, Michiels J. Formation, physiology, ecology, evolution and clinical importance of bacterial persisters. FEMS Microbiology Reviews. 2017;41(3):219–251. doi: 10.1093/femsre/fux001 28333307

[4] Balaban NQ, Merrin J, Chait R, Kowalik L, Leibler S. Bacterial Persistence as a Phenotypic Switch. Science. 2004;305(5690):1622–1625. doi: 10.1126/science.1099390 15308767

[5] Harms A, Maisonneuve E, Gerdes K. Mechanisms of bacterial persistence during stress and antibiotic exposure. Science. 2016;354(6318):aaf4268. doi: 10.1126/science.aaf4268 27980159

[6] Helaine S, Cheverton AM, Watson KG, Faure LM, Matthews SA, Holden DW. Internalization of *Salmonella* by Macrophages Induces Formation of Nonreplicating Persisters. Science. 2014;343(6167):204–208. 24408438 10.1126/science.1244705 PMC6485627

[7] Spoering AL, Lewis K. Biofilms and Planktonic Cells of *Pseudomonas aeruginosa* Have Similar Resistance to Killing by Antimicrobials. Journal of Bacteriology. 2001;183(23):6746–6751. 11698361 10.1128/JB.183.23.6746-6751.2001 PMC95513

[8] Leung V, Lévesque CM. A Stress-Inducible Quorum-Sensing Peptide Mediates the Formation of Persister Cells with Noninherited Multidrug Tolerance. Journal of Bacteriology. 2012;194(9):2265–2274. doi: 10.1128/JB.06707-11 22366415 PMC3347057

[9] Gutierrez A, Jain S, Bhargava P, Hamblin M, Lobritz MA, Collins JJ. Understanding and Sensitizing Density-Dependent Persistence to Quinolone Antibiotics. Molecular Cell. 2017;68(6):1147–1154.e1143. doi: 10.1016/j.molcel.2017.11.012 29225037

[10] Fisher RA, Gollan B, Helaine S. Persistent bacterial infections and persister cells. Nature Reviews Microbiology. 2017;15(8):453–464. doi: 10.1038/nrmicro.2017.42 28529326

[11] Mulcahy Lawrence R, Burns Jane L, Lory S, Lewis K. Emergence of *Pseudomonas aeruginosa* Strains Producing High Levels of Persister Cells in Patients with Cystic Fibrosis. Journal of Bacteriology. 2010;192(23):6191–6199. 20935098 10.1128/JB.01651-09 PMC2981199

[12] Griffin Amanda J, Li L-X, Voedisch S, Pabst O, McSorley Stephen J. Dissemination of Persistent Intestinal Bacteria via the Mesenteric Lymph Nodes Causes Typhoid Relapse. Infection and Immunity. 2011;79(4):1479–1488. doi: 10.1128/IAI.01033-10 21263018 PMC3067558

[13] Rossi O, Dybowski R, Maskell DJ, Grant AJ, Restif O, Mastroeni P. Within-host spatiotemporal dynamics of systemic *Salmonella* infection during and after antimicrobial treatment. Journal of Antimicrobial Chemotherapy. 2017;72(12):3390–3397. 28962012 10.1093/jac/dkx294 PMC5890750

[14] Arisoy AS, Altinişik B, Tünger Ö, Kurutepe S, Ispahi Ç. Maternal Carriage and AntimicrobialResistance Profile of Group B Streptococcus. Infection. 2003;31(4):244–246. doi: 10.1007/s15010-003-3182-6 14562949

[15] Le Doare K, Heath PT. An overview of global GBS epidemiology. Vaccine. 2013;31:D7–D12. doi: 10.1016/j.vaccine.2013.01.009 23973349

[16] Slotved H-C, Hoffmann S. The Epidemiology of Invasive Group B Streptococcus in Denmark From 2005 to 2018. Frontiers in Public Health. 2020;8:40. doi: 10.3389/fpubh.2020.00040 32211361 PMC7076979

[17] Vergnano S, Embleton N, Collinson A, Menson E, Russell AB, Heath P. Missed opportunities for preventing group B streptococcus infection. Archives of Disease in Childhood—Fetal and Neonatal Edition. 2010;95(1):F72–73. doi: 10.1136/adc.2009.160333 19439431

[18] Puopolo KM, Madoff LC, Eichenwald EC. Early-Onset Group B Streptococcal Disease in the Era of Maternal Screening. Pediatrics. 2005;115(5):1240–1246. doi: 10.1542/peds.2004-2275 15867030

[19] Jordan HT, Farley MM, Craig A, Mohle-Boetani J, Harrison LH, Petit S, et al. Revisiting the Need for Vaccine Prevention of Late-Onset Neonatal Group B Streptococcal Disease: A Multistate, Population-Based Analysis. The Pediatric Infectious Disease Journal. 2008;27(12):1057–1064. doi: 10.1097/INF.0b013e318180b3b9 18989238

[20] Wang Y-H, Chen H-M, Yang Y-H, Yang T-H, Teng C-H, Chen C-L, et al. Clinical and microbiological characteristics of recurrent group B streptococcal infection among non-pregnant adults. International Journal of Infectious Diseases. 2014;26:140–145. doi: 10.1016/j.ijid.2014.05.026 25058125

[21] Martins ER, Ó DNd, Costa ALM, Melo-Cristino J, Ramirez M. Characteristics of *Streptococcus agalactiae* Colonizing Nonpregnant Adults Support the Opportunistic Nature of Invasive Infections. Microbiology Spectrum. 2022;10(3):e01082–01022. 35604173 10.1128/spectrum.01082-22 PMC9241740

[22] van der Mee-Marquet N, Fourny L, Arnault L, Domelier A-S, Salloum M, Lartigue M-F, Quentin R. Molecular Characterization of Human-Colonizing *Streptococcus agalactiae* Strains Isolated from Throat, Skin, Anal Margin, and Genital Body Sites. Journal of Clinical Microbiology. 2008;46(9):2906–2911. 18632904 10.1128/JCM.00421-08 PMC2546740

[23] Green PA, Singh KV, Murray BE, Baker CJ. Recurrent group B streptococcal infections in infants: Clinical and microbiologic aspects. The Journal of Pediatrics. 1994;125(6, Part 1):931–938. doi: 10.1016/s0022-3476(05)82012-8 7996368

[24] Harrison LH, Ali A, Dwyer DM, Libonati JP, Reeves MW, Elliott JA, et al. Relapsing Invasive Group B Streptococcal Infection in Adults. Annals of Internal Medicine. 1995;123(6):421–427. doi: 10.7326/0003-4819-123-6-199509150-00004 7639441

[25] Ekelund K, Konradsen HB. Invasive group B streptococcal disease in infants: a 19-year nationwide study. Serotype distribution, incidence and recurrent infection. Epidemiology & Infection. 2004;132(6):1083–1090. 15635965 10.1017/s0950268804002808 PMC2870199

[26] Nielsen SY, Møller JK, Khalil MR. A comparison of GenomEra^®^ GBS PCR and GeneXpert ^®^ GBS PCR assays with culture of GBS performed with and without broth pre-enrichment. European Journal of Clinical Microbiology & Infectious Diseases. 2020;39(10):1945–1950. 32535806 10.1007/s10096-020-03934-4 PMC7497322

[27] Slotved H-C, Møller JK, Khalil MR, Nielsen SY. The serotype distribution of *Streptococcus agalactiae* (GBS) carriage isolates among pregnant women having risk factors for early-onset GBS disease: a comparative study with GBS causing invasive infections during the same period in Denmark. BMC Infectious Diseases. 2021;21(1):1129. 34724923 10.1186/s12879-021-06820-2 PMC8561911

[28] Lewis K. Persister Cells. Annual Review of Microbiology. 2010;64(1):357–372. 10.1146/annurev.micro.112408.134306 20528688

[29] Keren I, Kaldalu N, Spoering A, Wang Y, Lewis K. Persister cells and tolerance to antimicrobials. FEMS Microbiology Letters. 2004;230(1):13–18. doi: 10.1016/S0378-1097(03)00856-5 14734160

[30] Vega NM, Allison KR, Khalil AS, Collins JJ. Signaling-mediated bacterial persister formation. Nature Chemical Biology. 2012;8(5):431–433. doi: 10.1038/nchembio.915 22426114 PMC3329571

[31] Miki U, Miho F, Reiko O, Takashi N, Shoichi S, Hidenori N, et al. Observation of non-dormant persister cells reveals diverse modes of survival in antibiotic persistence. bioRxiv. 2021.10.28.466227.

[32] Jõers A, Tenson T. Growth resumption from stationary phase reveals memory in *Escherichia coli* cultures. Scientific Reports. 2016;6(1):24055. 27048851 10.1038/srep24055 PMC4822139

[33] Goneau Lee W, Yeoh Nigel S, MacDonald Kyle W, Cadieux Peter A, Burton Jeremy P, Razvi H, Reid G. Selective Target Inactivation Rather than Global Metabolic Dormancy Causes Antibiotic Tolerance in Uropathogens. Antimicrobial Agents and Chemotherapy. 2014;58(4):2089–2097. doi: 10.1128/AAC.02552-13 24449771 PMC4023725

[34] Hofsteenge N, van Nimwegen E, Silander OK. Quantitative analysis of persister fractions suggests different mechanisms of formation among environmental isolates of *E*. *coli*. BMC Microbiology. 2013;13(1):25. 23379956 10.1186/1471-2180-13-25 PMC3682893

[35] Mattiello SP, Barth VC, Scaria J, Ferreira CAS, Oliveira SD. Fluoroquinolone and beta-lactam antimicrobials induce different transcriptome profiles in *Salmonella enterica* persister cells. Scientific Reports. 2023;13(1):18696. 37907566 10.1038/s41598-023-46142-8 PMC10618250

[36] Willenborg J, Willms D, Bertram R, Goethe R, Valentin-Weigand P. Characterization of multi-drug tolerant persister cells in *Streptococcus suis*. BMC Microbiology. 2014;14(1):120. 24885389 10.1186/1471-2180-14-120 PMC4040513

[37] EUCAST. The European Committee on Antimicrobial Susceptibility Testing. Breakpoint tables for interpretation of MICs and zone diameters. 2021.

[38] Stewart B, Rozen DE. Genetic Variation for Antibiotic Persistence in *Escherichia coli*. Evolution. 2012;66(3):933–939. 22380453 10.1111/j.1558-5646.2011.01467.x

[39] Geerts N, De Vooght L, Passaris I, Delputte P, Van den Bergh B, Cos P. Antibiotic Tolerance Indicative of Persistence Is Pervasive among Clinical *Streptococcus pneumoniae* Isolates and Shows Strong Condition Dependence. Microbiology Spectrum. 2022;10(6):e02701–02722. 36374111 10.1128/spectrum.02701-22 PMC9769776

