# Supplementary figures and images for: Identification of antibiotic induced persister cells in Streptococcus agalactiae
Source: PLoS One. 2024 Jun 26;19(6):e0303271. doi: 10.1371/journal.pone.0303271 (PMC11207178; doi:10.1371/journal.pone.0303271)

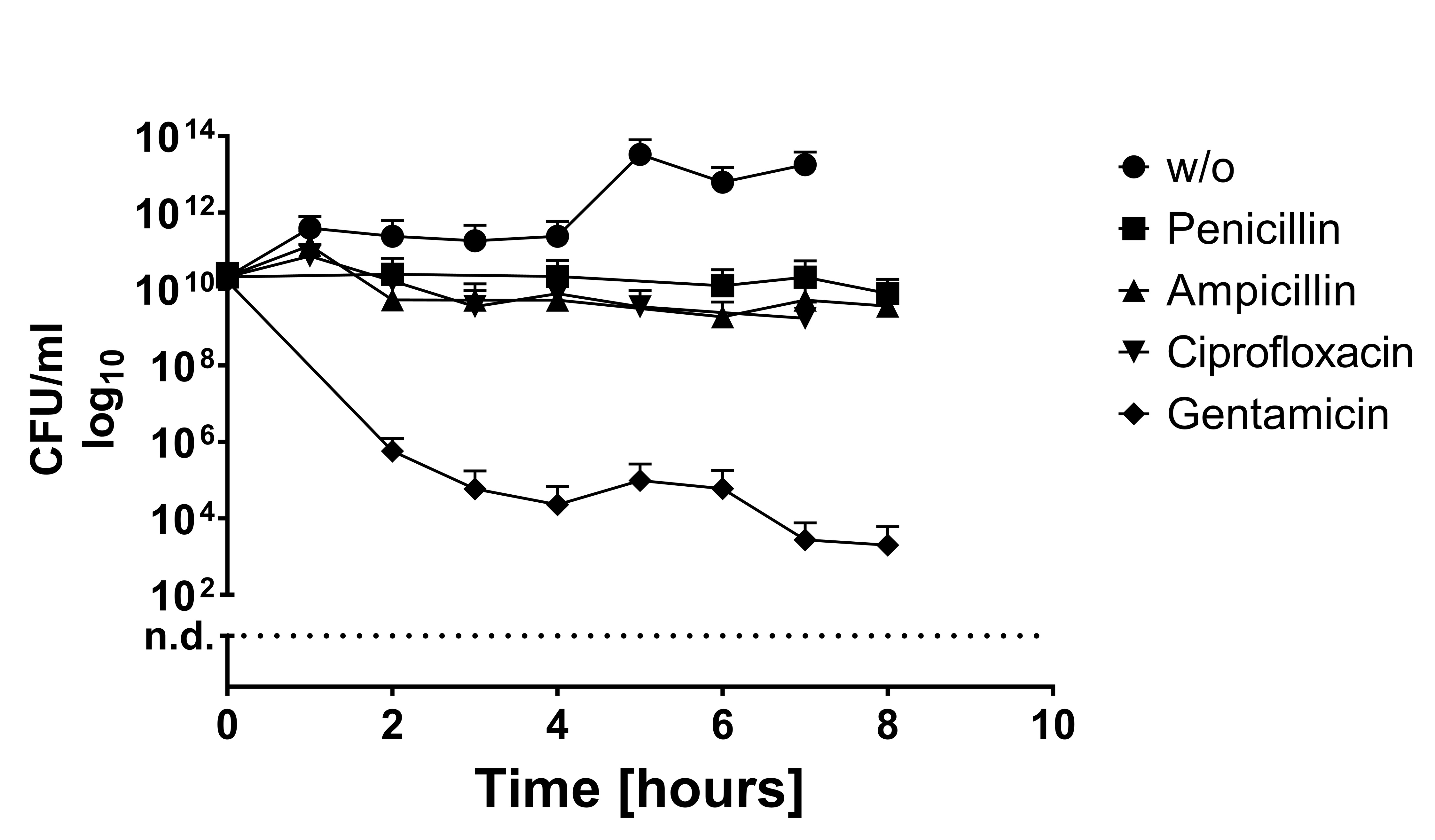

Supplement: S1 Fig — Late-exponential grown S. agalactiae strain NEM316 was challenged with 100-fold MIC of indicated antibiotics over time. The CFU/ml was calculated for each time-point, with a detection limit of 100 CFU/ml. The values are means of three biological replicates and error bars indicate the standard deviation. (TIF) [file pone.0303271.s001.tif]

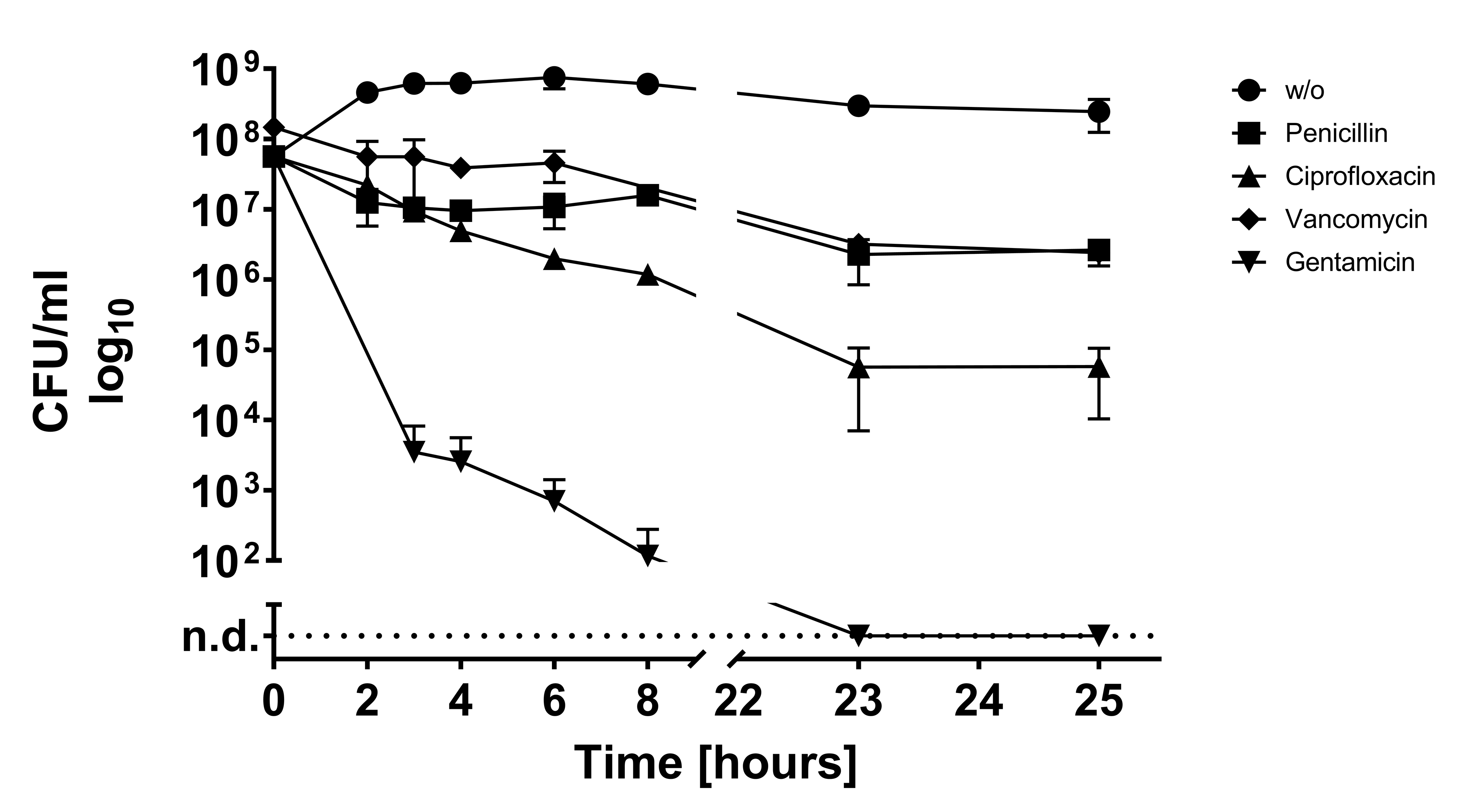

Supplement: S2 Fig — S. agalactiae strain NEM316 was grown to mid-exponential phase twice before challenged with 100-fold MIC of indicated antibiotics over time. The CFU/ml was calculated for each time-point, with a detection limit of 100 CFU/ml. The values are means of three biological replicates and error bars indicate the standard deviation. (TIF) [file pone.0303271.s002.tif]

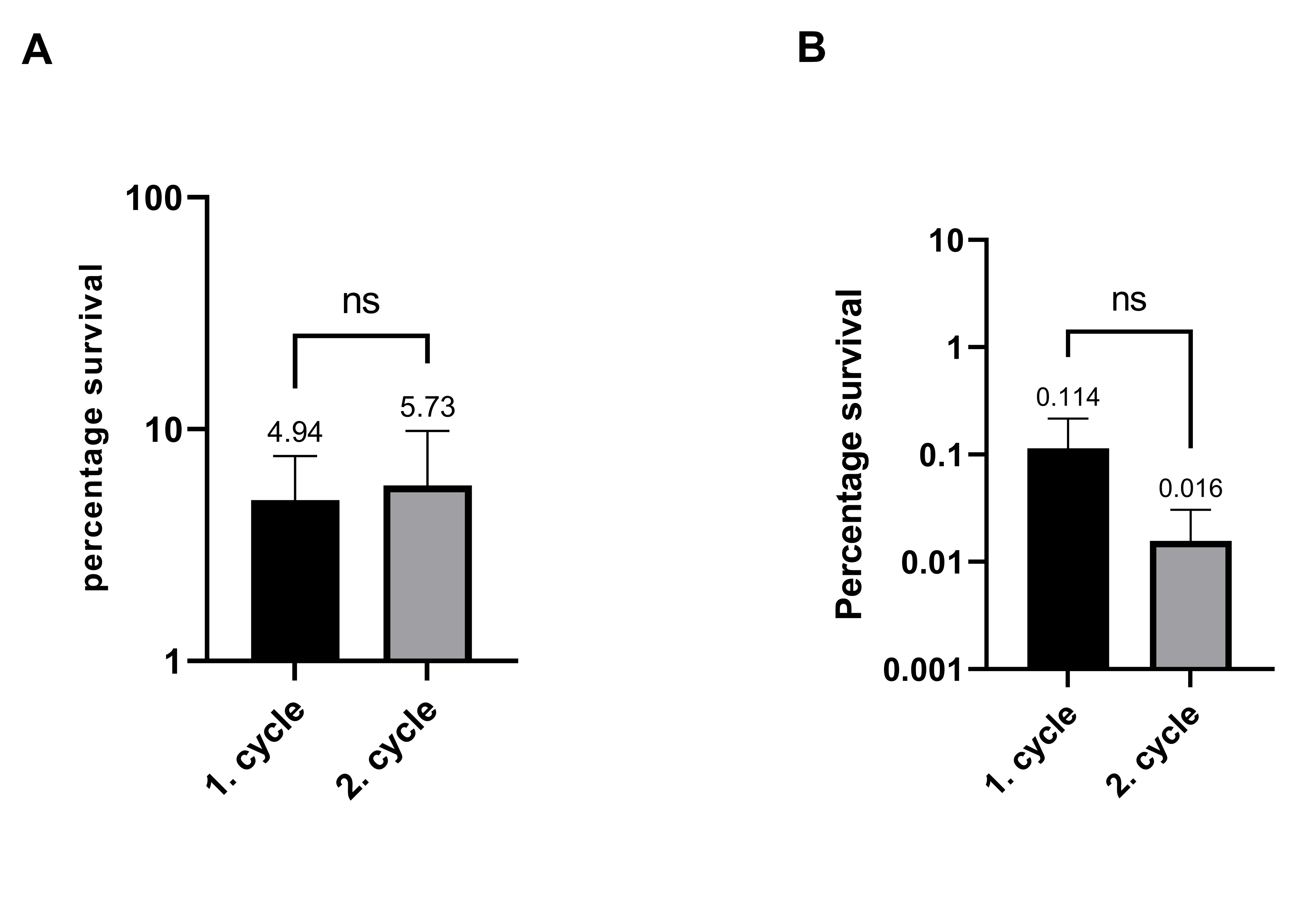

Supplement: S3 Fig — Surviving colonies from the first round of killing assay with (A) penicillin or (B) ciprofloxacin, has been exposed to a second round of killing using the same antibiotic. The persister cell fraction in percentage for each antibiotic after 25 hours are noted, and unpaired t-test was performed (P = <0.05), n.s. stands for not significant. The assay was performed in biological triplicates. (TIF) [file pone.0303271.s003.tif]

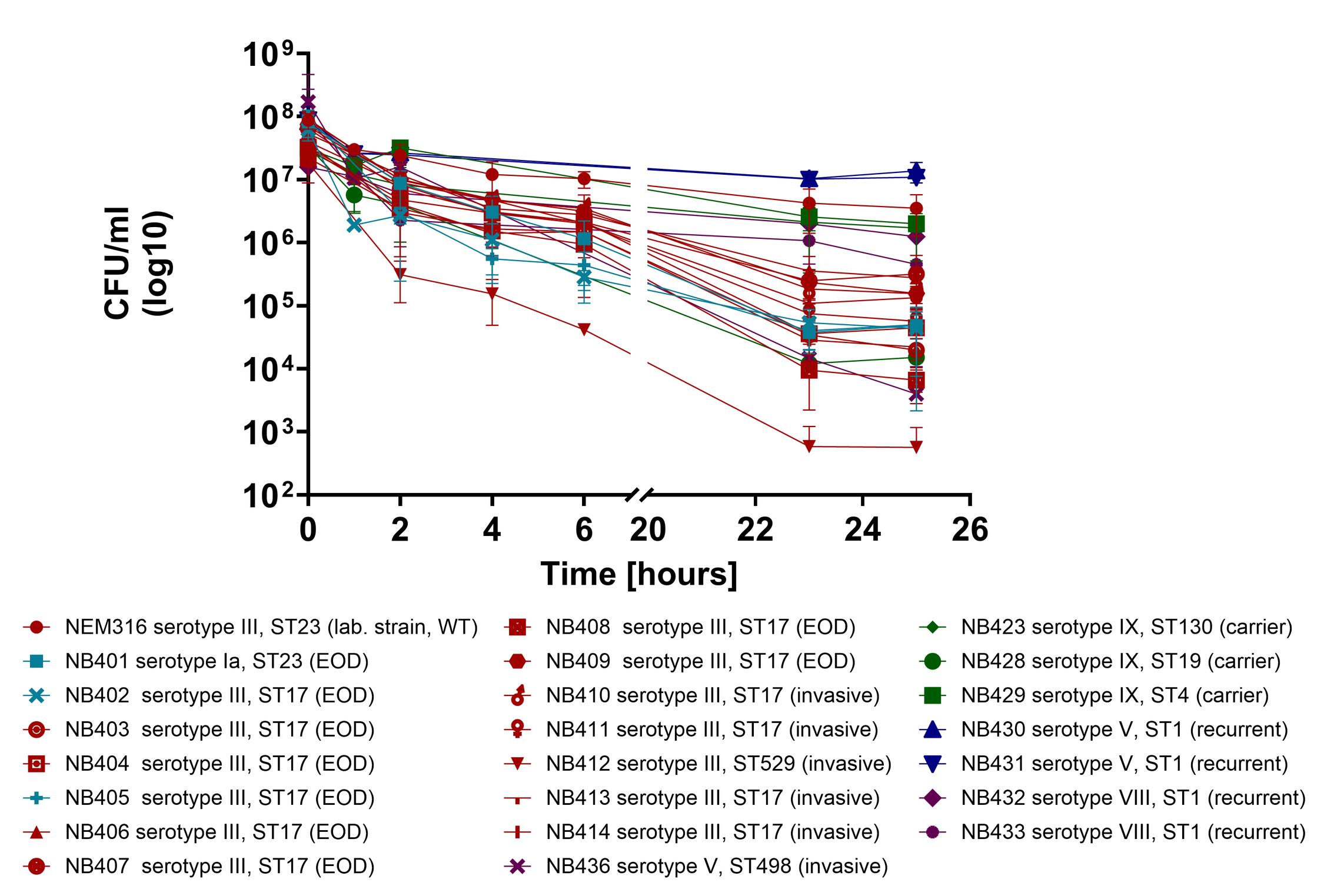

Supplement: S4 Fig — Clinical isolates of different S. agalactiae strains were grown to mid-exponential phase twice before challenged with 100-fold MIC of penicillin, and the CFU/ml was calculated for each time-point. The values are means of three biological replicates and error bars indicate the standard deviation. (TIF) [file pone.0303271.s004.tif]

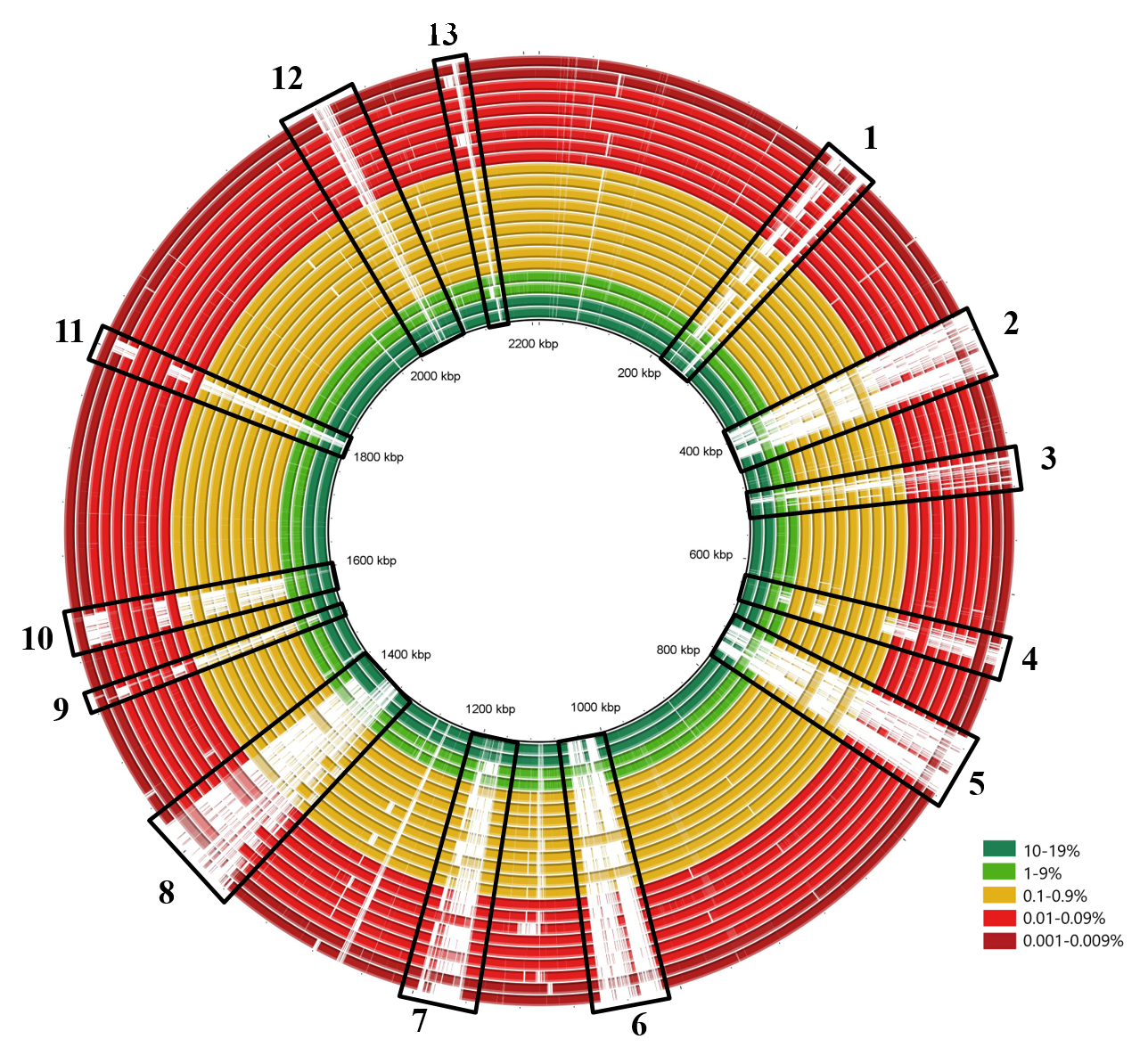

Supplement: S5 Fig — A comparative genomic analysis of all clinical isolates, organized according to persister levels. The central circle indicates the highest value, and the outer circles represent progressively lower values of persister frequency. Each circle is color-coded to reflect the persister level: Dark green for 10–19%, light green for 1–9%, yellow for 0.1–0.9%, light red for 0.01–0.09%, and dark red for 0.001–0.009%. The regions 1 to 13 highlight the areas with highest variations compared to NEM316. The figure is drawn by the Basic Local Alignment Search Tool (BLAST) Ring Image Generator (BRIG) using the genome of NEM316 as reference. (TIF) [file pone.0303271.s005.tif]
